# Supplementary material for: Establishing CREATE: lessons learned in setting up a training environment for early-career researchers in respiratory medicine
Source: BMC Med Educ. 2022 Mar 1;22:136. doi: 10.1186/s12909-022-03169-1 (PMC8886858; doi:10.1186/s12909-022-03169-1)
Supplement: Supplementary file 1 — Additional file 1: Supplementary Table 1. CREATE Professional Development Weekend – Program Overview. [file 12909_2022_3169_MOESM1_ESM.docx]

**Supplementary Table 1**

**CREATE Professional Development Weekend – Program Overview**

|  | **Friday** | **Saturday** | **Sunday** |
| --- | --- | --- | --- |
| **Morning** |  | Formal professional development sessions   - Specialist research content - Science for clinicians - Clinical update for scientists - Merging of scientific and clinical research - Strategic career planning, indicative topics: - Presenting a good track record - Planning an investigator-led research project/clinical trial - Using consumers in research - Grant writing   Ethics applications   - Building networks and collaborations | Presentations by ECRs - abstracts to be submitted |
| **Afternoon** | Arrive | Fun team building activities | Depart after lunch |
| **Evening** | Informal dinner with a “tangential” speaker | Formal dinner, attended by all plus industry sponsors |  |
